# Telepathology consultation for frozen section diagnosis in China

**DOI:** 10.1186/s13000-018-0705-0

**Published:** 2018-05-14

**Authors:** Yingxin Huang, Yan Lei, Qi Wang, Dazhou Li, Lili Ma, Lili Guo, Minshan Tang, Guanglong Liu, Qianwen Yan, Lan Shen, Guihui Tong, Zhiliang Jing, Yan Zhang, Yongjian Deng

**Affiliations:** Department of Pathology, Nanfang Hospital and School of Basic Medical Sciences, Southern Medical University, No.1838, Guangzhou North Road, Guangzhou, 510515 China

**Keywords:** Telepathology, Intraoperative consultation of pathology, Frozen section, Diagnosis

## Abstract

**Background:**

Telepathology (TP) provides remote pathology services for primary diagnosis practices, including intraoperative consultation of surgical pathology; it has not been widely implemented in China. In this study, the results of an implementation were reported, which lasted for two and a half years, and demonstrated the experience of the diagnosis of the intraoperative frozen sections by using TP consultation platform of Southern Medical University and Guangzhou Huayin Medical Laboratory Center (SMU-HUAYIN TP) in China.

**Methods:**

The SMU-HUAYIN TP consultation platform connects 71 participating basic hospitals and 11 senior pathologists. Nanfang Hospital is a high-level hospital located in a large city in China. This retrospective study summarizes the experience and results of TP for frozen section diagnosis by comparing the data of the platform and Nanfang Hospital over a period of 2.5 years from January 2015 to June 2017.

**Results:**

A total of 5233 cases were submitted to the platform, including 1019 cases in 2015, 2320 cases in 2016, and 1894 cases in 2017. The most common cases were breast (30.42%), followed by thyroid (29.05%) and gynecological (24.86%). Average turn-around time (TAT) of the cases from the platform in 2015 and 2016 was controlled within 30 min. In most TP cases (90.31%) and cases from Nanfang Hospital (86.14%), a definitive diagnosis was provided. The coincidence rate was 99.77% in the TP cases and 99.35% in the cases from Nanfang Hospital. The false positive and false negative rates of TP cases were 0.04 and 0.19%, respectively and no significant difference was found among different senior pathologists (*P* = 0.974, *P* = 0.989, *P* > 0.05). Similarly, there was no significant difference between TP cases and cases from Nanfang Hospital that were diagnosed by the same senior pathologist (*P* > 0.05).

**Conclusions:**

Our results indicate that TP in frozen section diagnosis could improve patient care and solve the problem of unevenly distributed pathology resources in China. We believe that in the near future, TP in frozen section diagnosis will become an important component of telemedicine and will play a significant role in health care reform in China.

## Background

Telepathology (TP), a terminology introduced in 1986 by Dr. Ronald Weinstein [1, 2], refers to the provision of pathology services over a distance using digital imaging processing and telecommunication. Certain pathological institutions or hospitals have used TP widely for routine surgical pathology [3–6], whereas others have used it more selectively for consultation with intraoperative frozen sections [7, 8].

The investment costs and time required to run a TP program are seen as barriers that prevent the widespread utilization of TP as an innovative standard technology for primary diagnostic practice, including diagnosis of intraoperative frozen sections [9, 10]. There has been a general perception that TP is unacceptably slow for contemporary diagnosis of intraoperative frozen sections. A number of pathologists have been of the opinion that the technique will be inaccurate and that poor image quality will result in deficient diagnoses. The latter were confuted by several retrospective validation studies that demonstrated an average accuracy rate of 95% for TP diagnoses of frozen sections [11–17]. The imaging technology used for TP has developed quickly. At present, TP has been transformed from static to whole slide imaging (WSI). WSI refers to the digitization of glass slides by scanners to produces images that simultaneously provides high resolution and a wide field of observations that can encompass the entire section, extending well beyond any single field of view.

Most TP studies to date have dealt more with the technological aspects than the clinical experience of this practice. Much of the clinical experience comes from the Western countries where the acceptance and utilization of TP have outpaced that in China. In this study, we share our experience of validating TP for intraoperative frozen section diagnosis in China.

## Methods

### Senior pathologists and hospitals

The SMU-HUAYIN diagnostic center was established by Southern Medical University in cooperation with HUAYIN Medical Laboratory Center, an independent medical laboratory in Guangzhou, China. The center is committed to promoting the development of TP in China and improving the pathological diagnosis level and clinical treatment in basic medical institutions. The center established a TP consultation platform in December 2014. By now, it has set up cooperative relationship with 71 basic hospitals without a senior pathologist on site. In this study, 5233 frozen TP cases were provided by the basic hospitals.

Nanfang Hospital is an affiliated hospital of Southern Medical University in Guangzhou, China. It is also a high level comprehensive teaching hospital with 2480 beds. In this study, 6205 frozen section cases were compared with the TP cases that were provided by Nanfang Hospital.

All of the senior pathologists came from Southern Medical University. Eight of them participated in the SMU-HUAYIN TP consultation platform. The other three pathologists took part in both the TP consultation platform and the Nanfang Hospital practice.

### Telepathology operation

**Fig. 1:**
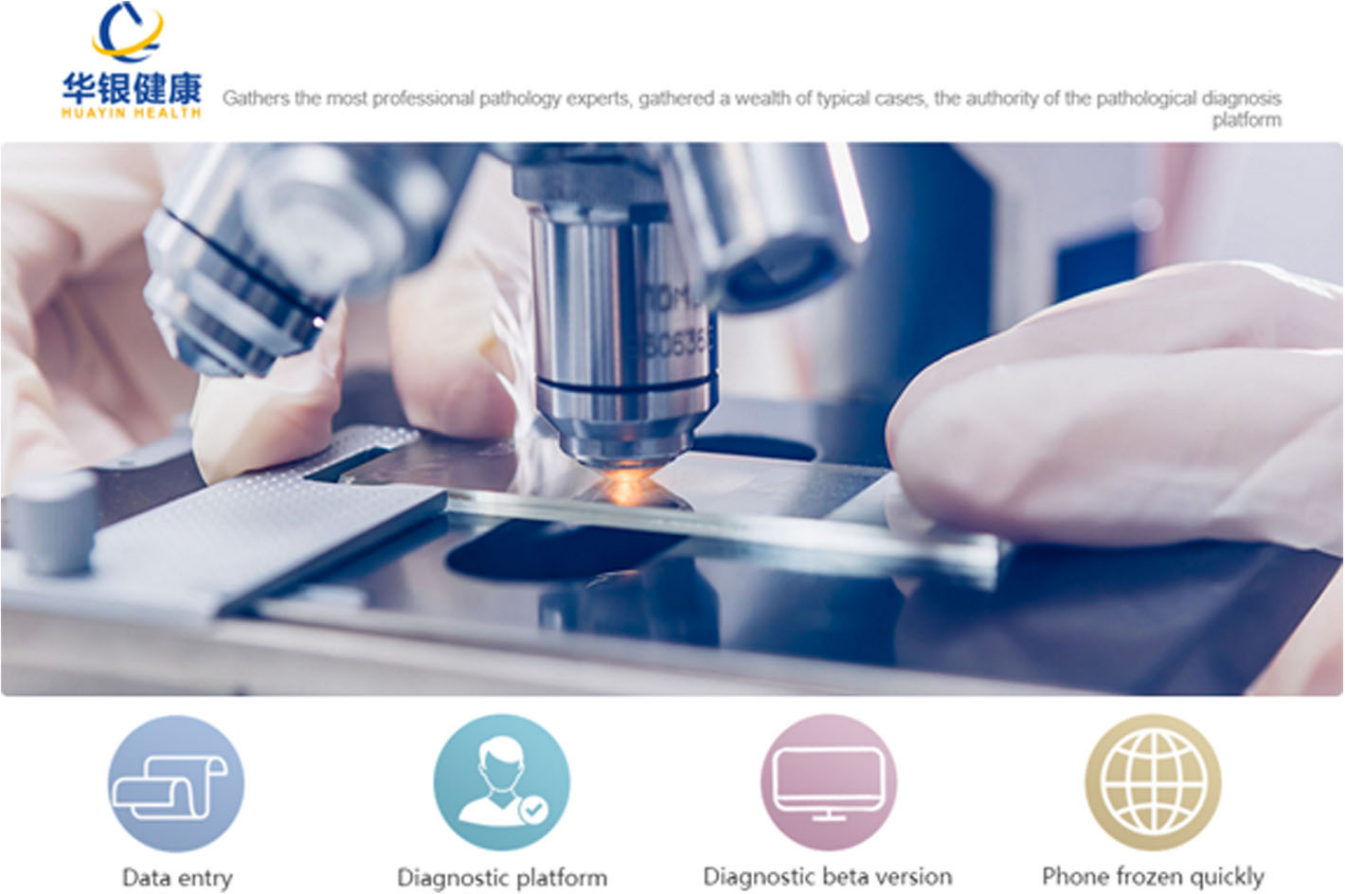
Customized SMU-HUAYIN TP consultation platform web portal. We have obtained permission from the copyright holders to reproduce the figure

A website based TP consultation platform, http://www.hypathology.com [18], was built to connect the basic hospitals and senior pathologists (Fig. 1). The pathologist residents on-site examine the surgical specimens, including taking photos, macroscopic description and sampling, they send gross photos and clinical information through the internet to the platform. When technologists finish making frozen sections, pathologists scan them into WSI and contact the senior pathologists by cell phone, informing them of pending consultation cases. Senior pathologists from SMU-HUAYIN diagnostic center log into the platform on a computer, check the pending cases and confirm the consultative diagnosis of pathology.

All 71 basic hospitals were equipped with a virtual microscope scanner (Motic EasyScan, China) which can scan frozen or routine histology glass slides and make digital images into a WSI. The microscope has 20× and 40× objectives. Senior pathologists from SMU-HUAYIN diagnostic center can browse the images (approximately 500 M of every slide) through the cloud data on the internet, and the scanning procedure could be completed within 10 min at an average network speed of 100 MB/s (40×, 15 mm × 15 mm).

### False positive diagnosis

Frozen sections were diagnosed as malignancy, while permanent sections were diagnosed as benign.

### False negative diagnosis

Frozen sections were diagnosed as benign, while permanent sections were diagnosed as malignant.

## Results

During the 2.5 year study period, 5233 cases were sent to the SMU-HUAYIN TP platform for consultation. There were 1019 cases in 2015, 2320 in 2016, and 1894 in 2017. An increasing number of basic hospitals have participated in the TP platform for frozen section consultation, wherein 27 in 2015, 54 in 2016, and 71 in 2017.

**Fig. 2:**
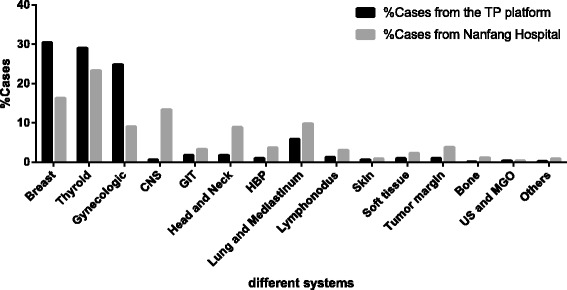
The distribution of cases among different systems.Abbreviations: CNS, Central nervous system; GIT, Gastrointestinal tract; HBP, Hepatobiliary and Pancreas; US, Urinary system; MGO, Male genital organs

Figure 2 showed the proportions of samples from systemic organs and tissues. In all TP cases, the most cases came from the breast(30.42%), followed by thyroid (29.05%) and gynecological organs (24.86%). The largest proportion of frozen samples from Nanfang Hospital was thyroid (23.27%),followed by breast (16.29%) and central nervous system (13.34%).

Turn-around time (TAT) was counted as the time when pathologists in basic hospitals received the intraoperative specimens to the time when senior pathologists produce the report with a final diagnostic. 5101 cases of the TP platform and 6186 cases of Nanfang Hospital, respectively, were sent to TAT analysis in this study. Table 1 shows that the average TAT of TP cases was 28.6 min in 2015 and 30.7 min in 2016, while the TAT of frozen section cases from Nanfang Hospital was 28.5 min in 2015 and 32.2 min in 2016. The average TAT of TP cases in 2015 and 2016 was controlled within 30 min. In 5101 cases of the TP platform, 3058 cases took less than 30 min, 1809 cases between 30 and 60 min, and 234 cases more than 60 min. By reviewing those cases costing more than 60 min, the common causes are: 1) surgeons submitting the specimens once again, 2) appending sampling and scanning repeatedly, 3) abnormalities of computer, scanner or network connection.

**Table 1:** TAT comparison between cases from the TP platform and from Nanfang Hospital

	2015 TAT(min)	2016 TAT(min)	Total TAT(min)
Cases from the TP platform	28.6	30.7	29.7
Cases from Nanfang Hospital	28.5	32.2	30.3

Based on the diagnoses rendered by senior pathologists, the cases were divided into two groups: those with confirmed diagnoses (benign or malignant) and those with unconfirmed diagnoses (deferred to permanent). Confirmed diagnoses were provided in 90.31% of TP cases and 86.14% of cases of Nangfang Hospital (Table 2). The unconfirmed cases were most commonly associated with the limitation of frozen sections and were not attributable to the digitization of frozen slices. The limitation of frozen sections has been a factor in 37.28% of our unconfirmed TP cases. A typical example would involve the follicular tumor of the thyroid that needs to be widely sampled to observe whether there is capsular or vascular invasion in order to judge if the tumor is benign or malignant. Another example is that those cannot be made into frozen section biopsy, such as calcified, ossified and adipose tissues.

**Table 2:** Concordance data comparison between cases from the TP platform and from Nanfang Hospital

Feature	TP platform (%)	Nanfang Hospital (%)
Confirmed	4726 (90.31)	5345 (86.14)
concordance	4715 (99.77)	4710 (99.35)
False positive	2 (0.04)	3 (0.06)
False negative	9 (0.19)	28 (0.59)
Unconfirmed	507 (9.69)	860 (13.86)
Total	5233	6205

Pathologists from basic hospitals performed the last gross examination of the specimens, thawed the frozen tissue and sent the WSI of permanent sections to the senior pathologists to make the final diagnoses under optical microscopy. All 4726 confirmed TP cases had a corresponding permanent section diagnosis with a 0.04% false positive rate and a 0.19% false negative rate (Table 2). A detailed description of false positive and false negative cases from the TP platform is outlined in Table 3. In 5345 confirmed cases from Nanfang Hospital, 4741 cases had a corresponding permanent section diagnosis with a 0.06% false positive rate and a 0.59% false negative rate (Table 2). Of the 4726 confirmed TP cases, concordance between frozen section and permanent section diagnoses was noted in 4715 (99.77%) cases. A similar concordance rate was recorded for the frozen section cases (99.35%) (Table 2). Table 4 shows the level of concordance for the diagnosis of breast (100%), thyroid (99.46%) and gynecological (99.92%) samples in TP cases.

**Table 3:** In concordance cases of intraoperative frozen sections from the TP platform

	Lesion site	Telepathological diagnosis	Final diagnosis (permanent)	Annotation
False positive:				
Cases 1	Right lung	Malignant tumor	Sclerosing pneumocytoma	N/A
Cases 2	Right thyroid	Diffuse infiltrating follicular carcinoma	Nodular goiter with adenomatous hyperplasia	N/A
False negative:				
Cases 1	Parotid gland	Consistent with benign tumors and considered as lymphoid adenoma	Low-grade mucoepidermoid carcinoma	N/A
Cases 2	Right thyroid	Nodular goiter with cystic degeneration and partial follicular epithelial hyperplasia actively	Nodular goiter with papillary carcinoma (3 mm in diameter)	Cancer failed to be sampled
Cases 3	Left thyroid	Benign nodules	Follicular carcinoma of thyroid with minimal capsular invasiveness, eosinophilic subtype	Failed to sample the invasive lesion
Cases 4	Left thyroid	Nodular goiter with calcification	Papillary carcinoma	Calcified cancer failed to be sliced
Cases 5	Right ovary	Benign cystic lesion	Serous borderline ovarian tumor with severe atypical hyperplasia	Failed to borderline lesion
Cases 6	Right thyroid	Nodular goiter	Nodular goiter with localized papillary carcinoma (1.3 mm in diameter)	Cancer failed to be sampled
Cases 7	Right thyroid	Nodular goiter	Nodular goiter with localized papillary carcinoma	Cancer failed to be sampled
Cases 8	Left thyroid	Nodular goiter with fibrosis and ossification	Nodular goiter with fibrosis and papillary carcinoma	Fibrosis and ossification unable to be sliced well
Cases 9	Left thyroid	Nodular goiter	Papillary thyroid carcinoma	Cancer failed to be sampled

**Table 4:** The comparison of concordance rate in different body systems between cases from the TP platform and from Nanfang Hospital

Organ	TP platform		Nanfang Hospital	
	Cases	%	Cases	%
Breast				
Total	1487		816	
concordance	1487	100	816	100
Thyroid				
Total	1306		1132	
concordance	1299	99.46	1113	98.32
Gynecologic				
Total	1204		347	
concordance	1203	99.92	347	100

The false negative rate and false positive rate of different senior pathologists in the SMU-HUAYIN TP consultation platform are shown in Table 5. There was no significant difference in false negative rates and false positive rates between different senior pathologists (*P* = 0.974, *P* = 0.989, *P* > 0.05). Table 5 also shows the false negative rate and false positive rate of three senior pathologists working in the SMU-HUAYIN TP consultation platform and Nanfang Hospital. There was no significant difference in false negative rates and false positive rates between TP cases and frozen section cases that were diagnosed by the same senior pathologist (*P* > 0.05).

**Table 5:** The false negative rate (FNR) and false positive rate (FPR) of senior pathologists in the TP platform and Nanfang Hospital

Senior pathologist	Cases from the TP platform					Cases from Nanfang Hospital				
	FP cases	FN cases	Confirmed cases	FPR	FNR	FP cases	FN cases	Confirmed cases	FPR	FNR
A	1	5	1986	0.05%	0.25%	2	7	1007	0.20%	0.70%
B	0	1	809	0.00%	0.12%	0	1	723	0.00%	0.14%
C	1	1	667	0.15%	0.15%	0	2	430	0.00%	0.47%
D	0	0	322	0.00%	0.00%					
E	0	1	220	0.00%	0.45%					
F	0	0	311	0.00%	0.00%					
G	0	0	14	0.00%	0.00%					
H	0	0	25	0.00%	0.00%					
I	0	1	255	0.00%	0.39%					
J	0	0	24	0.00%	0.00%					
K	0	0	7	0.00%	0.00%					
Total	2	9	4640	0.04%	0.19%	2	10	2160	0.09%	0.46%

## Discussion

There have been limited studies about the use of TP in frozen section diagnosis in China. Several published articles reported a government supported TP consultation and quality control program for cancer diagnosis in China, from which indicated that TP could play an important role in pathology diagnosis for China healthcare services [19–21]. In recent years, TP has been applied successfully in routine surgical pathology consultation. To date, no large cohort assessment has been shown to prove the successful application of TP in frozen section diagnosis in China.

Our experience of 2.5-year remote pathological practice has been shown to attract basic hospitals to participate in the frozen TP consultation platform. Quality control of TP meets the needs of medical services so that the basic hospitals submit more cases to the platform for frozen TP consultation. This finding suggested that TP in frozen section diagnosis is increasingly accepted and should be implemented widely in the developing countries just as China.

Breast, thyroid and gynecologic cases accounted for the largest proportion and the high concordance rate in TP cases. Our results indicated that TP in frozen section diagnosis could help the surgical department in basic hospitals, especially in specialties such as general surgery and gynecology, to perform evidence-based surgeries, optimize the operation plan and avoid additional operation effectively. Analysis of the TAT of TP diagnosis in frozen sections showed that the average time spent in our TP reports was within 30 min, which conforms to the intraoperative diagnostic criteria for frozen sections in China.

A report from the University Health Network in Toronto showed that the concordance rate and the definitive rate in frozen section diagnosis using TP was 98 and 92.3%, respectively [9]. Similar to those in Toronto, our result from 4726 TP cases that had corresponding permanent section diagnosis showed that the concordance rate was 99.77%, and the definitive rate was 90.31% in 5233 TP cases. However, the concordance rate and definitive rate in the frozen section cases from Nanfang Hospital was 99.35 and 86.14%, respectively, which were less than that of our TP cases. Quality control in histopathology is quite important that all the phases of the process leading to a histological report should be adequately controlled [22]. Our results indicated that TP has yielded high-quality frozen section diagnosis in China.

The success of TP in frozen section diagnosis was dependent on low operating cost, use of simple technologies, bi-directional communication, strong team leadership, IT expertise, appropriate training, and user acceptance. Hospitals in China are divided into three levels: Level 3 hospitals are large hospitals with comprehensive medical, teaching, research abilities that are located in big cities, Level 2 hospitals are mid-sized hospitals that are located in small to middle sized cities, Level 1 hospitals are those located in the countryside or small cities. According to the statistics of the National Ministry of Health, there are only 9841 licensed pathologists in China, and nearly 70% of them are concentrated in the Level 3 hospitals. If every 100 hospital beds should have 1–2 pathologists, 40,000–90,000 pathologists are urgently needed.

In addition, there is a notable shortage of senior pathologists who can independently complete the diagnosis of frozen sections in basic hospitals, making it difficult for basic hospitals to perform surgical procedures. TP in frozen section diagnosis is helpful in alleviating the shortage of senior pathologists in basic hospitals and supporting the surgical services of these hospitals. For basic hospitals with small number of frozen specimens, using TP platform is much less costly than hiring a senior pathologist to residents on-site. In the future, TP or digital pathology will play a pivotal role in frozen section diagnosis and quality control, as its current development is accelerated by the advance of digital and internet technology and is supported by the government. TP in frozen section diagnosis also benefits patients so that they no longer need to wait for the diagnosis with paraffin sections, thereby saving time and avoiding reoperation. We believe that in the near future, TP in frozen diagnosis will become an important component of telemedicine and will play a significant role in health care reform in China.

## Conclusions

In summary, our retrospective review showed that quality control of the TP intraoperative consultation was equal to the on-site work. TP in frozen section diagnosis could play an important role in improving patient care and solving the shortage of pathology resources in China. Therefore, TP in frozen section diagnosis should be encouraged and actively undertaken.

## Abbreviations

CNS: Central nervous system
GIT: Gastrointestinal tract
HBP: Hepatobiliary and pancreas
MGO: Male genital organs
TAT: turn-around time
TP: Telepathology
US: Urinary system
WSI: Whole slide imaging
